# Swallowing and QoL Outcomes, Patient Experience and Treatment Related Priorities in Recurrent Oropharyngeal Cancer (rOPC)- a Mixed Method Study

**DOI:** 10.1007/s00455-025-10898-0

**Published:** 2025-12-04

**Authors:** Grainne Brady, Justin Roe, Vinidh Paleri, Pernilla Lagergren, Mary Wells

**Affiliations:** 1 The Royal Marsden NHS Foundation Trust, London, UK; 2 Department of Surgery & Cancer, Imperial College London, London, UK; 3 Imperial College Healthcare Trust, London, UK; 4 Department of Molecular Medicine and Surgery, Karolinska Institutet, Stockholm, Sweden

**Keywords:** Recurrent oropharyngeal cancer, Swallowing, Quality of life, Treatment decision-making

## Abstract

**Background:**

Recurrent oropharyngeal cancer (rOPC) presents challenging treatment decision-making. Toxicity from previous treatment, coupled with potential functional and quality of life (QoL) morbidity of further treatment(s) can influence decision-making regarding curative and non-curative treatment.

**Aim:**

To investigate swallowing and QoL outcomes, treatment-related priorities, and patient experience before and after rOPC treatment.

**Method:**

Longitudinal, multicentre, mixed method study. Outcomes included the MD Anderson Dysphagia Inventory (MDADI), Performance Status Scale for Head and Neck Cancer (PSS-HN), the University of Washington QoL Questionnaire (UWQoLv4), the Chicago Priority Scale (CPS) and semi-structured interviews.

**Results:**

The sample included 37 participants (prospective *n* = 25, retrospective *n* = 12, females *n* = 6) with a median age of 63 (range 41–88). The majority (*n* = 21) had curative intent treatment (surgery *n* = 20 or radiation *n* = 1). The remainder had non-curative intent immunotherapy (*n* = 14) or chemotherapy (*n* = 2). Swallowing and QoL were impaired at baseline; median MDADI composite score: 60 (IQR: 52.15–81.75), median PSS- HN normalcy of diet: (NoD): 50 and UWQoLv4 global health score (GHS): 60 (IQR 40–60). The PSS-HN NoD score deteriorated to 40 at six months. The MDADI and UWQoL data remained impaired. Triangulation with qualitative data revealed agreement with the PSS data and provided context for the relatively stable QoL. Being cured of cancer and living as long as possible remained the key priorities at all timepoints.

**Conclusion:**

In this study swallowing deteriorated with treatment for rOPC. Treatment-related priorities remained focused on cure or survival. If the treatment-related goal was achieved, patients adapted and experienced at least stable QoL regardless of swallowing status.

**Supplementary Information:**

The online version contains supplementary material available at 10.1007/s00455-025-10898-0.

## Background

Despite ongoing treatment advances in primary oropharyngeal cancer (OPC) aiming to increase cure rates, optimise swallowing and quality of life (QoL) outcomes, a proportion of patients continue to experience recurrence. Locoregional recurrent oropharyngeal cancer (rOPC) rates have been reported at 9% for human papilloma virus (HPV)-positive disease and 26% for HPV- negative disease [1]. Disease which recurs within 12 months of previous radiation treatment is referred to as residual disease, between 12 months and five years as recurrent disease and more than five years is typically considered as second primary disease [2]. Frequently, patients will have had previous radiation as part of their initial treatment for OPC [2]. In this setting, residual, recurrent and new primary disease categories can be considered as a homogenous group, due to the technical difficulties involved in re-challenging treatments (complex surgery/re-irradiation) and potential baseline functional difficulties and/or persisting or late toxicities due to previous radiation exposure [3].

Historically, patients with recurrent head and neck cancer (rHNC) had a very poor prognosis with the majority of patients receiving best supportive care [4]. However, over the past two decades there have been rapid advances in the treatment of recurrent disease in particular for rOPC. A landmark systematic review and meta-analysis investigated survival rates for those undergoing curative treatments including surgery and re-irradiation [5]. This study revealed a 5-year survival rate of just 18% for those treated curatively prior to the year 2000, which increased to 51% following the year 2000. This has led to a paradigm shift in the management of rOPC with more patients being considered for curative treatments including surgery or re-irradiation [4]. In parallel, there have also been significant changes in the treatment of patients who have inoperable rHNC, where re-irradiation is not feasible (non-curable disease). Historically these patients, if medically fit enough, would have been offered either chemotherapy with an average prognosis of approximately eight months [6], or best supportive care [4]. Two landmark trials KEYNOTE 040 [7] and KEYNOTE 048 [8] have resulted in the adoption of immunotherapy as standard of care for patients with inoperable disease due to an observed clinically meaningful survival benefit with immunotherapy over standard chemotherapy. Further long term follow up has also shown a lasting response with 30% survival at four years in very selected patients based on the biological markers of their tumours [9]. Whilst both of these trials looked at rHNC including multiple subtypes, a large proportion of the sample included patients with rOPC. Collectively, the research in curative and non-curative treatments for rOPC has completely changed the landscape of management. Indeed, treatments continue to evolve, and multiple concurrent investigations of novel therapeutics for patients with rHNC are currently underway with some promising early results [10].

Treatment decision-making in the context of recurrent disease presents some of the most challenging management issues in HNC surgical and oncological practice [4]. Baseline function and QoL difficulties confound an already complex decision-making process when it comes to choosing one treatment over another [3]. Quite often patients may need to make the difficult decision to pursue potential cure over the ability to maintain eating and drinking and/or speaking. Curative treatment options pose a high risk of complications, high rates of feeding tube dependence (where eating/drinking is no longer possible) and poor speech outcomes [11]. A careful balance between the desire to achieve cure and maintain function and QoL is critical to the decision between curative or non-curative treatments vs. clinical trials and may be based on the potential functional morbidity of these choices [3]. Given that patients with rOPC have previous/current experience of function and QoL change due to their previous treatment(s) for primary disease, it is important not only to quantify further change during and after salvage or palliative treatment(s) using multidimensional assessment including patient reported outcome measures. It is also imperative to contextualise such changes with how patients experience and prioritise these changes over time.

The aim of this study was to identify and explore function and QoL outcomes, patient priorities and patient experience of rOPC over time including diagnosis, treatment decision-making and living with the consequences of treatment.

## Materials and Methods

### Design

A convergent mixed method prospective and retrospective study was designed [12]. The prospective arm involved the collection of both qualitative and quantitative data. The retrospective arm involved the collection of qualitative data only. The qualitative and quantitative data were collected concurrently, analysed separately and then combined via integration.

Setting: Two specialist cancer centres in the United Kingdom (UK).

Ethical considerations: The research protocol was underpinned by Patient and Public Involvement and Engagement (PPI/E) from concept to completion. Study protocol was approved by the Royal Marsden NHS Foundation Trust Committee for Clinical Research (CCR5242) and the Health Research Authority (HRA) (IRAS: 297893).

Participants: Patients with biopsy proven loco-regionally residual, recurrent, or new primary OPC in a previously irradiated field who were undergoing or had previously undergone treatment for their recurrence, including surgery, re-irradiation immunotherapy, chemotherapy or a clinical trial of an investigational agent were included in the study. Inclusion/exclusion criteria are listed in Table 1.

**Table 1:** Inclusion exclusion criteria

Inclusion criteria	Exclusion criteria
• Adult (aged 18 years or over) competent to provide consent • Have a diagnosis of rOPC - Prospective group: newly diagnosed - Retrospective group: at least 12 months post diagnosis • Have adequate linguistic and cognitive function to participate to complete questionnaires. • English speaking	• Any previous medical condition, other than HNC, which has a known impact on communication/swallowing (Parkinson’s Disease, Multiple Sclerosis, cerebral vascular accident (CVA)

### Sampling

The overall sampling design for the longitudinal mixed methods study was multi-level [13]. For the qualitative workstream, purposive sampling was utilised to ensure a deep insight into the experience of rOPC was gained across curative and non-curative pathways. For the quantitative workstream a probability-based sample size calculation was undertaken for the primary outcome measure.

### Recruitment

Eligible patients were identified by their usual care team and provided with study information at their standard of care outpatient appointments. They were contacted by lead author GB to confirm participation/recruitment. Recruitment was competed across two urban sites, one in London and one in Newcastle.

### Data Collection

Function and QoL data were collected prospectively using a range of patient reported and clinician-rated function and QoL measures including the MD Anderson Dysphagia Inventory (MDADI) [14], the Performance Status Scale for Head and Neck Cancer (PSS-HN) [15] and the University of Washington Quality of Life version 4 (UWQoLv4) questionnaire [16]. Gastrostomy tube usage was also recorded. The timepoints for collection of these measures included baseline prior to treatment initiation, and three and six months following treatment initiation.

Patient priorities were measured using the Chicago Priority Scale (CPS) [17] at baseline and six months. Concurrent qualitative data was collected using semi structured interviews. For the participants these data were collected longitudinally at baseline prior to treatment initiation and at six months following treatment initiation. For the retrospective sample, qualitative data were collected at a single timepoint, at least 12 months or more following treatment initiation for recurrent disease. The qualitative data study has been described in detail in a previous publication [18].

### Data Analysis

Qualitative data: Interviews were audio recorded and transcribed verbatim. Data analysis was completed using Framework Approach (FA) [19]. NVivo software for Mac (version 20.7.1 Lumivero, Denver United States of America) was used to support the analysis.

Quantitative data: The purpose of this study was exploratory, and although a sample size calculation was completed in advance, the study was underpowered, therefore the analysis was descriptive. IBM SPSS Statistics Version 29.0.1.0 (171) was used in the quantitative data analysis. Baseline patient characteristics (age, gender, ethnicity and previous HNC treatment history) were summarised in addition to the diagnostic (site and time of recurrence) and treatment details (type of treatment). Outcome measures including the MDADI, PSS-HN NoD and gastrostomy were described in terms of medians and interquartile range (IQR) at baseline, three and six months. The CPS data was described using medians and IQRs at baseline and six months.

Integration: As a mixed methods study, integration of the quantitative and qualitative aspects was planned at the stages of design, methods and interpretation to enhance the results gained and maximise the advantages of both quantitative and qualitative data [20]. The combined use of joint display tables (see supplementary file for example) with a triangulation protocol fitted the research question and also ensured equity across the two data sets, in keeping with the mixed method methodology. In addition to the visual display of integration, via joint tables, and the triangulation protocol to identify agreement, partial agreement, discordance and silence, it was also felt that narrative integration would be required, where the quantitative and qualitative findings are described thematically in relation to the research questions for this study. The combination of joint tables and narrative description has been reported in the literature in particular with previous convergent mixed methods studies [20].

## Results

### Participants

In total 37 participants were included, with 25 who were recruited prospectively and 12 who were recruited retrospectively. The majority of participants were male, with a median age of 64 years (range 41–88) in the prospective group and 64 (range 59–79) in the retrospective group. All of the patients except one had previously been treated for primary disease with radiation +/- chemotherapy. One patient had been previously treated with surgery alone. Demographics for sample are summarised in Table 2. Due to sample attrition due to death, only 21/25 prospective participants were followed up at six months. The results of this study therefore only relate to the participants who survived at least six months following diagnosis of recurrence.

**Table 2:** Participant demographics

		Prospective *n* = 25	Retrospective *n* = 12
**Sex**	Male	20	11
	Female	5	1
**Ethnicity**	White British	22	11
	Mixed- White & Black African	1	0
	Asian	2	1
**Age**	Median	64	64
	Range	41–88	59–70
**Recurrent disease classification**	Residual disease (diagnosed within 12 months of primary treatment)	5	1
	Recurrent disease (diagnosed 12 months – 5 years following treatment for primary disease)	12	8
	New primary disease (diagnosed more than 5 years following treatment for primary disease).	8	3
**Treatment type**	Curative	14	7
	Non-curative	11	5
**Previous radiation treatment**	Yes	24	12
	No	1	0

### Quantitative Findings

#### Swallowing Outcomes

**Fig. 1:**
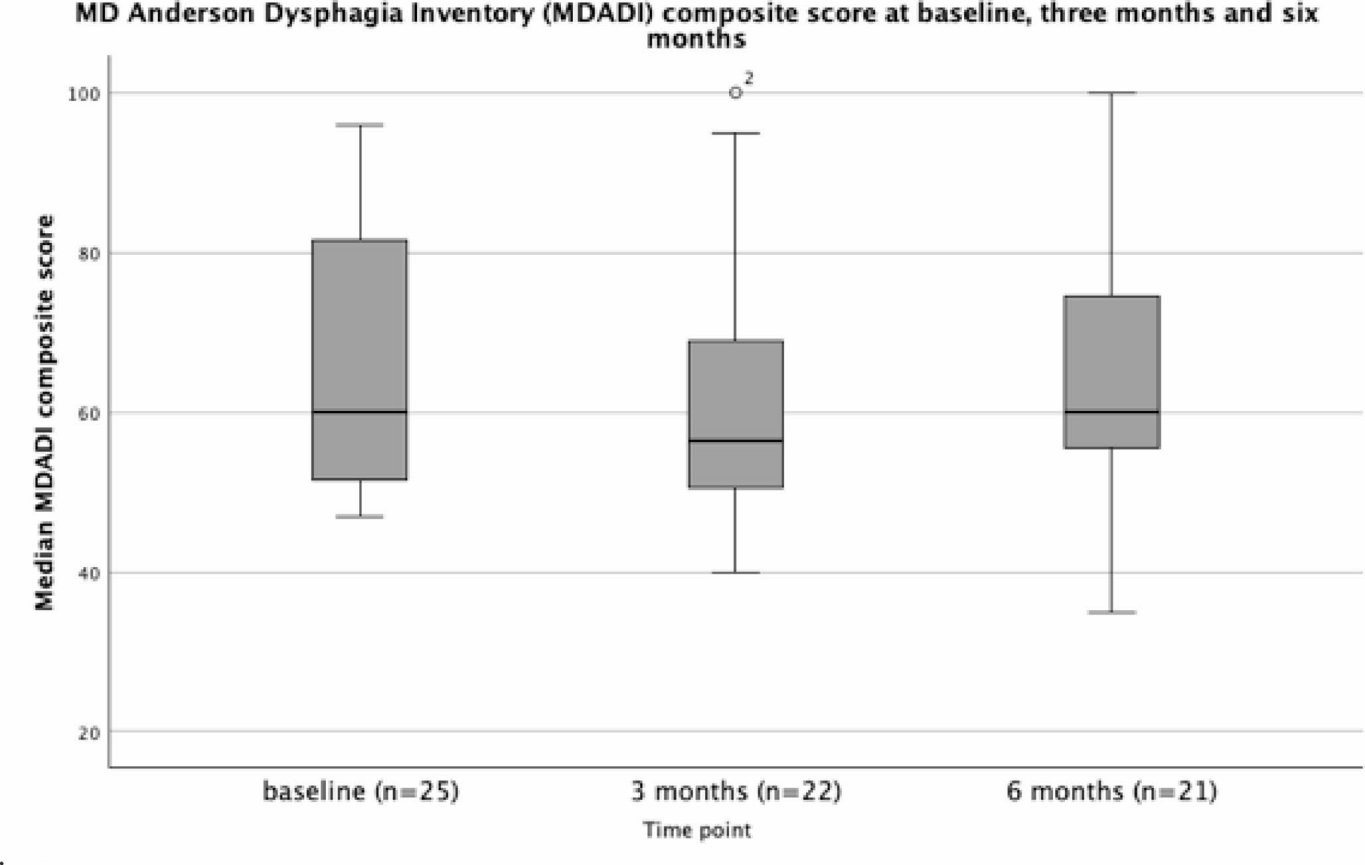
MD Anderson Dysphagia Inventory (MDADI) composite scores at baseline prior to treatment initiation, and three and six months following treatment initiation. Scores range from 20–100 where 20 is the worst possible score. Scores of ≥ 80 are considered optimal, ≥ 60-<80 adequate,<60 poor. At each timepoint the horizontal line represents the median, the box represents the interquartile range and the whiskers demonstrate the minimum and maximum values. Outliers are marked with a O

The MDADI scores range from 20 to 100 where 20 is the worst possible score. Scores of ≥ 80 are considered optimal, ≥ 60-<80 adequate,<60 poor. The baseline MDADI composite score for the sample was 60 (IQR: 52.15–81.75), at three months this deteriorated to 56.50 (IQR 49.75- 72.0) returning to 60 (IQR 54.75–75.75) at six months (Fig. 1).

The PSS-HN NoD Scores range from 0 to 100 where 0 is the worst possible score (nil by mouth) and 100 is the highest possible score (normal diet with no restrictions). Impaired diet scores were noted using the PSS-HN NoD data at baseline with a deterioration across timepoints as summarised in Table 3. At baseline there were three participants who were unable to eat/drink prior to commencing treatment, this increased to six at three months and reduced to five at six months following treatment initiation. Increased diet modification was noted across treatment groups, with an increase in participants requiring pureed/non-chew diet from two at baseline to seven at six months.

**Table 3:** Performance status scale for head and neck cancer (PSS-HN) normalcy of diet (NoD) frequency and median scores at baseline, three months and six months

Timepoint		Baseline			3 months			6 months		
Score	Description	Curative (*n* = 14)	Non-curative (*n* = 11)	**Total** (*n* = 25)	Curative (*n* = 13)	Non curative (*n* = 10)	**Total** (*n* = 23)	Curative (*n* = 13)	Non-curative (*n* = 8)	**Total** (*n* = 21)
100	Full diet (no restrictions)	1	0	**1**	1	0	**2**	1	1	**2**
90	Full diet (liquid assist)	4	3	**7**	2	1	**3**	4	0	**4**
80	All meats	1	0	**1**			**0**			**0**
70	Carrots, celery			**0**			**0**			**0**
60	Dry bread and crackers	1	1	**2**			**0**			**0**
50	Soft chewable foods	4	4	**8**	3	4	**7**	0	1	**1**
40	Soft foods requiring no chewing			**1**	1	0	**1**	2	3	**5**
30	Pureed foods	1	1	**1**	1	1	**2**	1	1	**2**
20	Warm liquids	1		**1**	1	0	**1**			**0**
10	Cold liquids			**0**	2	0	**2**	2	0	**2**
0	Non-oral feeding only	1	2	**3**	2	4	**6**	3	2	**5**
**Median**		**50**			**40**			**40**		

**Fig. 2:**
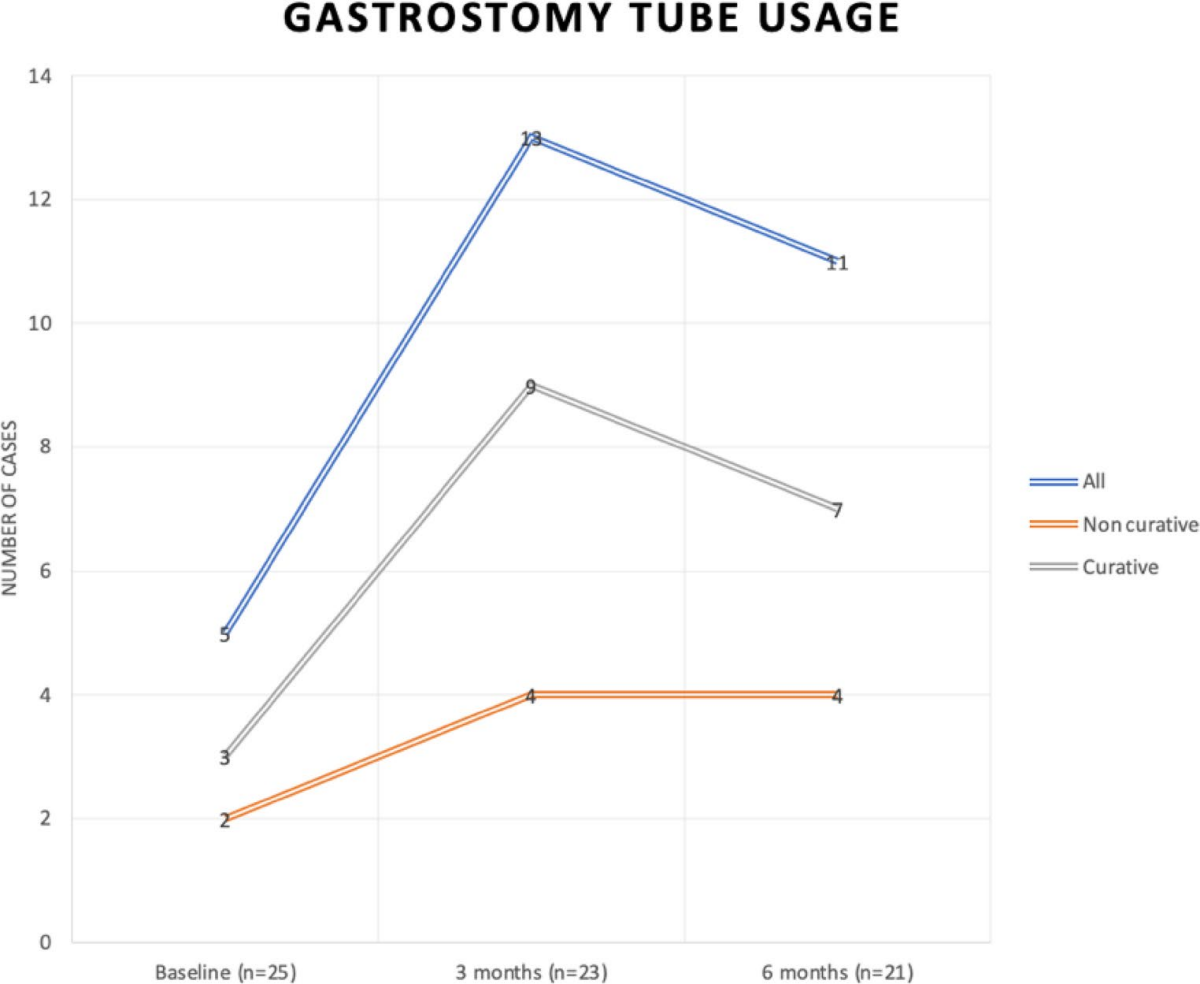
Gastrostomy tube usage at baseline, three and six months. The blue line represents the sample, and the grey and orange line represent the curative and non-curative treatment group participants

Gastrostomy tube usage was observed to increase for the entire sample at three months, remain stable at six months for the non-curative group but decrease for the curative group, as seen in Fig. 2.

#### Quality of Life Outcomes

**Fig. 3:**
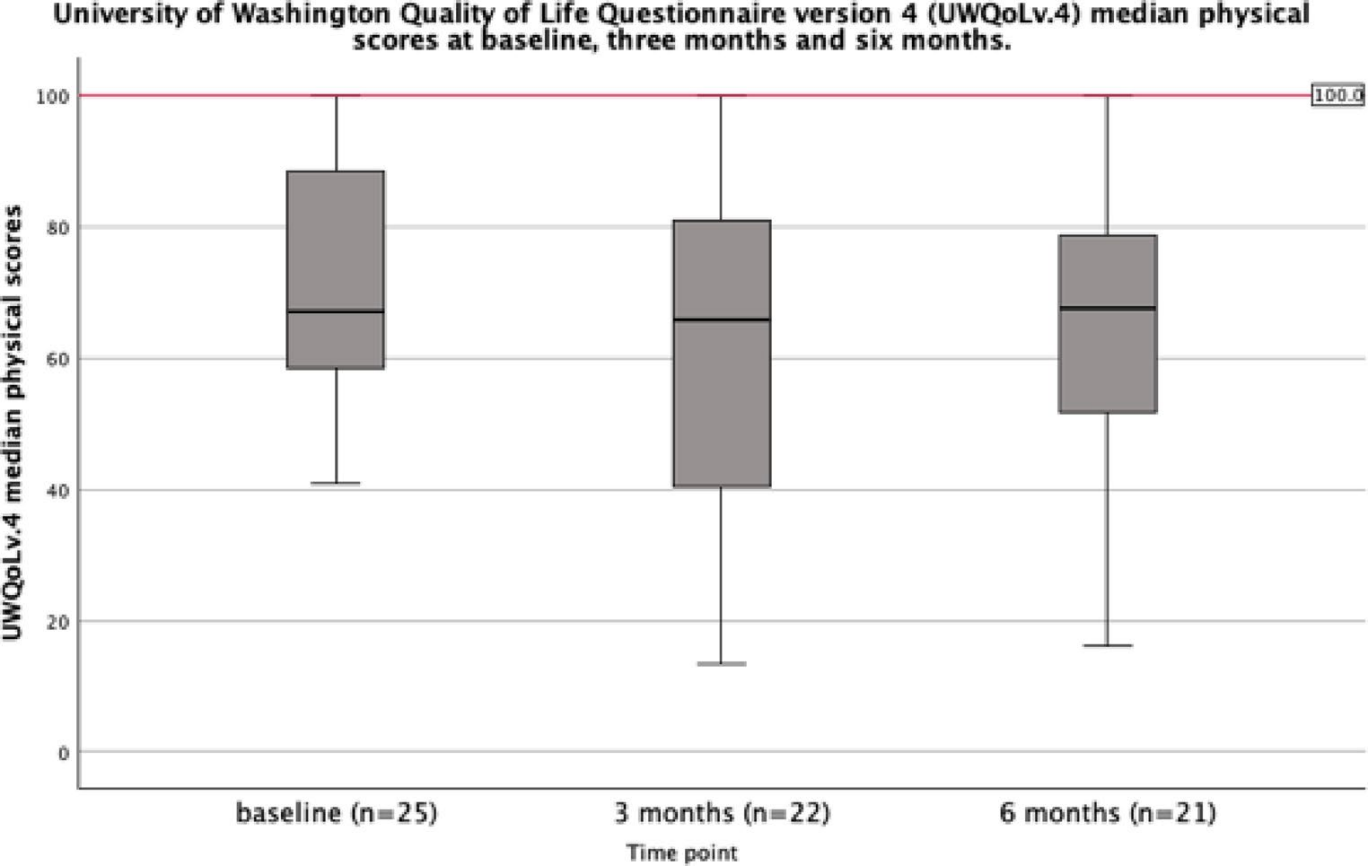
University of Washington quality of life questionnaire v.4 physical composite scores at baseline before treatment initiation, and three and six months following treatment initiation

The median UWQoLv4 physical score was 68 (IQR: 58–88) at baseline, 64 (IQR 41–80) at three months, and 63 (IQR 46–78) at six months. A mild deterioration in physical composite score is noted between baseline and three months, and three months and six months as summarised in Fig. 3.

**Fig. 4:**
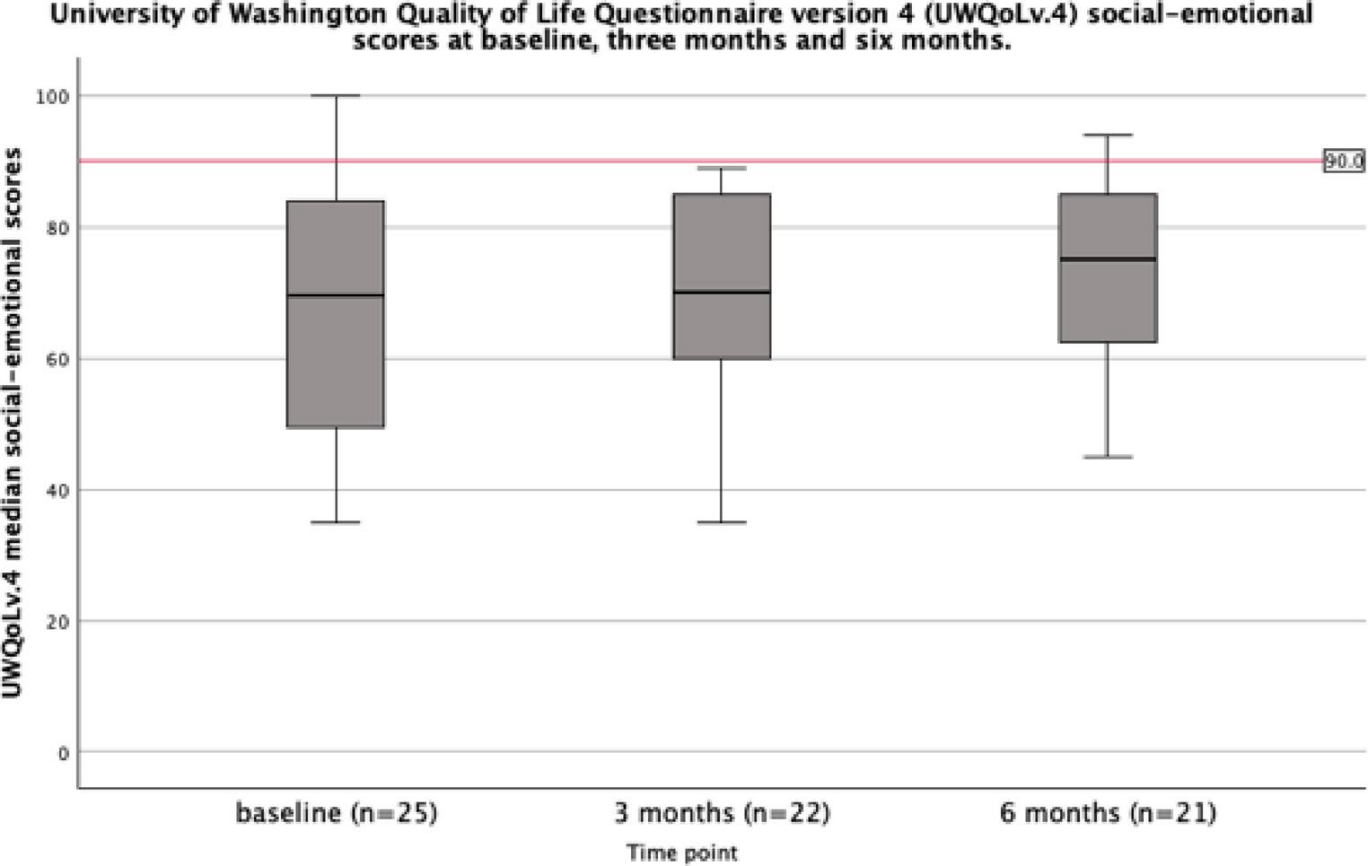
University of Washington Quality of Life Questionnaire v.4 physical composite scores at baseline before treatment initiation, and three and six months following treatment initiation

The median UWQoLv4 social-emotional score was 70 (IQR: 50–82) at baseline and remained stable at 70 (IQR 54.5–85) at three months and improved to 74 (IQR 58–85) at six months, as summarised in Fig. 4.

#### Treatment Related Priorities

The CPS was completed by 24/25 participants at baseline and 20/21 at six months. One participant declined to fill out this questionnaire at both timepoints. Being cured of cancer, living as long as possible and being pain free were the top three priorities at baseline and six months both within and across treatment pathways.

### Qualitative Findings

The qualitative findings have been detailed in a previous publication [18]. As described previously, the patient experience data which emerged from the interview data revealed three main themes. The first theme entitled ‘Here we go again’ refers to the participants’ experience of a recurrent diagnosis in the context of their previous experience of primary OPC. Participants set the scene with their description of their previous experience of primary OPC diagnosis, treatment and recovery after treatment. The second theme entitled ‘do I have a choice’ describes participants’ experiences of how they ended up on a particular treatment pathway, including access to second opinions and under involvement or otherwise in decision-making. The third and final theme ‘A personal choice’ relates to living with and beyond the diagnosis and treatment of disease recurrence and finding balance between survival, overall QoL and functional status in both the curative and non-curative treatment pathways. Participants contextualise their experience of recurrent disease in their past experience of primary disease diagnosis and treatment. Patients want to survive and when the options to choose between are cure or functional outcomes impacting on health related QoL, cure appears to be favoured. However, when cure is not an option, patients appear to want to survive as long as possible. Patients do experience a deterioration in function over time, however they do appear to be able to adapt and maintain QoL once their overall goal of cure or prolonged survival is being achieved. However, as the prognosis gets shorter there appears to be a shift in priorities where function/QoL take precedence over survival.

### Integrated Findings

#### Patient Experience

When patients are diagnosed with disease recurrence, one of their greatest fears, the fear of recurrence, becomes a reality. These patients used their experience of HNC to frame how they responded to the recurrence at symptom presentation, diagnosis, treatment and through survivorship. The overarching findings of this mixed methods research are that although there may be a common treatment, common outcomes and common goal of cure/survival, each person experiences this differently. For each person, every effort must be made to enhance that individual’s experience of the care they are receiving. At the point of diagnosis and treatment decision making, and at every juncture in care thereafter, there must at least be the option for shared decision making.

#### Swallowing and QoL

Although we identified a discordance between patient-reported outcome measures and the clinician rated measures of swallowing in this study, our overall integrated findings identified that swallowing is impaired at baseline and is likely to deteriorate over time. Patients are aware of their impaired function at baseline and of the likely decline over time.

QoL is also often impaired prior to treatment initiation and is likely to change after treatment for rOPC. Aspects of quality of life are at risk of deterioration. However, some aspects of QoL may improve with treatment for rOPC. To find balance between the sacrifices made to achieve cure or prolonged survival and the deterioration in function and QoL, patients appear to be able to recalibrate what QoL is and means to them.

#### Treatment-related Priorities

Treatment-related priorities did not change in our sample and remained focused on cure or survival, with patients acknowledging lower success rates and the possibility of poorer function/QoL. If a less invasive option is available and will not compromise their overall survival, patients will choose this. Patients’ treatment-related priorities do not appear to change over time if their disease remains stable. Cure/survival remain key whatever the cost in terms of function/QoL. However there does appear to be some more consideration for QoL, if and when a patient’s circumstances change. This was seen in the non-curative setting over time at junctures between treatment options, or when curative treatment was not successful. It seems that when the prognostic outlook begins to shorten, more consideration is given to function and QoL.

## Discussion

The aim of this mixed method study was to measure function and QoL outcomes, priorities and patient experiences of rOPC. This aim was addressed using a mixed method convergent design including a repeated measures observational quantitative workstream and a longitudinal qualitative workstream involving interviews with patients. Although a small sample of patients were included, limited to two recruitment sites in the UK, this study has, to our knowledge, offered the most detailed account of information in relation to patient experience of OPC recurrence diagnosis, treatment decision making, treatment and living with and beyond treatment. The main findings of the qualitative workstream of this study have been published in a separate publication [18], and the findings of the integration of these qualitative findings with the quantitative findings are presented here. Although the protocol for analysis and integration of the findings was prespecified, allowing transparency and rigour of the research method, there remains a risk of bias. The lead author GB is an experienced clinician and has worked in the field of rOPC for many years which may have influenced the interpretation and integration of the qualitative and quantitative findings. The potential for bias is a limitation of this study.

The primary outcome in the quantitative workstream, the MDADI, demonstrated no change in swallowing from baseline before treatment to six months following treatment initiation. This particular outcome measure, the MDADI, is widely used in the HNC research as a primary or secondary outcome measure in many large international trials [21, 22]. In line with best practice guidance for the conduct of HNC research [23], multi-faceted measures of swallowing were also used in the quantitative workstream, including a number of secondary outcome measures. The secondary outcome measures focusing on function (swallowing) were clinician-rated measures. In this study there was discordance between the primary and secondary outcome measures in relation to swallowing function. Secondary endpoints including the PSS-NoD and gastrostomy tube usage suggested a deterioration in swallowing function. A discordance in patient and clinician rated outcomes has been highlighted previously in primary HNC research. However, in the primary setting, it has been found that clinicians can underestimate the level of impairment [24]. Consequently, there is an emphasis in both clinical practice and research to utilise patient reported- outcome measures [25]. In this study however, if we looked at the primary outcome measure of the quantitative workstream in isolation, one might disregard the secondary outcome findings and interpret no change in swallowing as demonstrated by the MDADI. However, as per the aim of this mixed method study, we have a deeper understanding of swallowing from the qualitative data. In this study, the qualitative workstream data demonstrates disagreement with the patient-reported swallowing outcome but is more concordant with the clinician-rated outcomes showing a decline in swallowing. The content validity of the MDADI has more recently come under scrutiny, in particular for those patients with a greater level of dysphagia severity [26] and those with HPV-related OPC [27] and so this may offer some explanation to the findings presented.

With regards to QoL, the quantitative data has shown that some elements of QoL, namely the physical domains, decline over time, in particular for those undergoing curative treatment. Agreement was noted with the qualitative data, with many participants describing that they had experienced changes in their physical status and ability to participate in activities of daily living. Those who underwent curative treatment described a sudden deterioration in their physical functioning followed by a degree of improvement which did not reach their baseline level of functioning. For some this may be an improvement in strength, energy levels or mobility. For others this may be gradual improvement in swallowing. The trajectory of change in QoL was not the same for participants on a non-curative treatment pathway. Overall, from baseline prior to treatment initiation to six months following treatment initiation, a mild improvement in physical functioning was noted. This is in part explained via the qualitative findings, where participants report some symptomatic relief with treatment commencement or when symptoms such as pain were addressed.

An area of common QoL improvement noted quantitatively from baseline to six months in the entire sample and across treatment pathways was in the social-emotional QoL scale. One might not expect this in either the curative or non-curative setting given the perceived potential functional/QoL morbidity with curative treatment, or the palliative diagnosis associated with non-curative treatment. The qualitative data in this study provides further explanation as to why participants may experience improvements in some aspects of social-emotional domains of QoL. Patients, particularly in the curative group, appear to experience a phenomenon known as ‘response shift’ where patients experience better outcomes over time, not because the patient is physically doing better (as we can see from the deterioration in physical QoL related score) but because the patient has adapted to their new circumstances [28].

The overarching finding of this mixed method study is the importance of patient experience. It is not possible to separate treatment-related priorities, function and QoL, from patient experience. Clinicians should consider treatment-related priorities in the context of patient experience. Rather than asking ourselves if we know what the patient wants, we need to ensure that the patient has been given adequate and appropriate information to understand their treatment options. Healthcare professionals need to ensure that patients have the opportunity to make their priorities heard and be involved in decision making. Establishing patients’ experience of function and QoL before, during and after treatment is crucial before informing them of functional and QoL changes that can happen with treatment. Clinicians should appraise patients of the direction in which function and QoL is likely to change and when this is likely to happen. For each individual patient, every effort must be made to enhance that individual’s experience of the care they are receiving. Being diagnosed with rOPC is a significant life event, however this does not preclude a positive patient experience. In the qualitative arm of this study which was previously published [18], we have heard accounts where patients were alone at the time the diagnosis was delivered. Although second opinions were accessed by some patients, the pathway to accessing a second opinion was not always explicit or understood by both patients and healthcare professionals. There were very mixed reports regarding patient involvement in decision-making, with some experiencing a lack of involvement or a feeling of being pushed towards a particular or sole treatment route. The experience of pre-treatment counselling also varied greatly, with some reporting how ill-prepared they felt before their treatment, whilst others, many of whom admitted they felt overwhelmed at the time, could see the benefits of the preparations following the event.

## Conclusion

This study has provided new knowledge that has the potential to influence how treatment decisions are made not for but with this patient population. It is imperative that healthcare professionals identify the key treatment-related goal for the patient, and this must be acknowledged when discussing all other relevant clinical information in treatment planning. Given the patients’ level of experience with HNC treatment and its aftermath, this experience must be harnessed so that patients can truly envision the potential scenarios with various treatment options in keeping with true shared decision making. This will ultimately influence the informed consent process and pre-treatment counselling for all patients with rOPC.

## Supplementary Information

Below is the link to the electronic supplementary material.

Supplementary Material 1

## Data Availability

All data supporting the findings of this study are available within the paper and its Supplementary Information.
