# Supplementary material for: Swallowing and QoL Outcomes, Patient Experience and Treatment Related Priorities in Recurrent Oropharyngeal Cancer (rOPC)- a Mixed Method Study
Source: Dysphagia. 2025 Dec 4;41(2):473–82. doi: 10.1007/s00455-025-10898-0 (PMC13099690; doi:10.1007/s00455-025-10898-0)
Supplement: Supplementary file 1 — Supplementary Material 1 [file 455_2025_10898_MOESM1_ESM.docx]

Supplementary file:

Joint display table for qualitative and quantitative findings regarding swallowing at timepoint one prior to treatment initiation

| Main theme | Qualitative findings | Quantitative findings1 |
| --- | --- | --- |
| Here we go again-  Something not quite right | Participants were aware of their baseline swallowing difficulties. Quite often further deterioration in swallowing or discomfort on swallowing was the presenting symptom of their disease recurrence. For those participants who were diagnosed with residual/ early recurrent disease, ongoing toxicity from their previous treatment made it difficult for some participants to decipher between persisting toxicities and emerging symptoms indicative of new disease.  When participants voiced their concern regarding swallowing it didn’t necessarily relate to the enjoyment of food, they were concerned for example that they would not be strong enough to fight the disease, or in the setting of a prophylactic gastrostomy pre- surgery, to have to live with a gastrostomy long term. | MDADI ([Chen et al., 2001](#_ENREF_16)) composite score: 60 (IQR: 52.15-81.75)  Median PDD-HN NoD ([List et al., 1990](#_ENREF_74)) score: 50  Gastrostomy tube: 20%  UWQoLv4 ([Rogers et al., 2002](#_ENREF_127)): swallowing included in top 3 concerns: yes  CPS ([Sharp et al., 1999](#_ENREF_137)): swallowing ranked 4/12 in order of priority |
| Gerard and his wife Sophie | Gerard: ‘[Swallowing]…It’s fine. I mean I just had dinner tonight, that was a bit of a struggle eating the broccoli and that. Sometimes it’s fine and then, other days, you feel like it’s sort of sticking at the back of your throat’. | MDADI composite score: 84  PSS-HN NoD: 90  Gastrostomy: no  Swallowing in top three concerns on UWQoLv4: No  CPS: swallowing ranked 5/12 |
| Toby | ‘The biggest reason I’d like to get rid of the RIG, and I know I need to be able to eat and drink before I do, is it doesn’t seem normal. It makes me feel like there is something wrong with me, which I know there is at the moment, but it would be nice to jump out of bed every morning and not see it, like, you know? And then consequently, if the RIG wasn’t there, I’d get back to doing things that I think the RIG might impose on like at the gym. But I’m big enough to admit that if I can’t drink and I can’t swallow, the RIG is going to keep me alive’. | MDADI composite: 60 (adequate)  PSS-HN NoD: 50 (soft food)  Gastrostomy: yes (prophylactic)  Swallowing in top three concerns on UWQoLv4: Yes  CPS: swallowing ranked 4/12 |
| Kim | ‘My swallowing is a pain in the bum because [before my first treatment] I did enjoy my food and it was like a social thing… but not anymore…’ | MDADI composite: 50 (poor)  PSS-HN NoD: 20 (hot and cold liquids)  Gastrostomy: yes  Swallowing in top three concerns on UWQoLv4: No  CPS: swallowing ranked 3/12 |
| Triangulation: partial agreement, participants had baseline swallowing difficulties, and it was one of the top symptoms concerns however, was not one of the top three treatment-related priorities for most. | | |
